# Increasing the data on elasmobranch plasma protein electrophoresis: electrophoretogram reference values determination in the undulate skate (Raja Undulata) and the nursehound shark (Scyliorhinus stellaris) maintained under human care

**DOI:** 10.1186/s12917-022-03478-z

**Published:** 2022-10-29

**Authors:** Pablo Morón-Elorza, Carlos Rojo-Solís, Christine Steyrer, Teresa Álvaro-Álvarez, Mónica Valls-Torres, Javier Ortega, Teresa Encinas, Daniel García-Párraga

**Affiliations:** 1 Department of Pharmacology and Toxicology, Faculty of Veterinary Medicine, Complutense University of Madrid, Av. Puerta de Hierro s/n, 28040 Madrid, Spain; 2 Fundación Oceanogràfic de la Comunitat Valenciana, C/ Eduardo Primo Yúfera (Científic) 1B, 46013 Valencia, Pablo, Spain; 3 Veterinary Services, Carlos Rojo-Solís, Teresa Álvaro-Álvarez, Mónica Valls-Torres, Oceanogràfic. Ciudad de las Artes y las Ciencias. C/ Eduardo Primo Yúfera (Científic), 1B, 46013 Valencia, Spain; 4 Lionsrock Big Cat Sanctuary, Bethlehem, South Africa; 5 VISAVET Health Surveillance Centre, Complutense University of Madrid, Madrid, Spain; 6 Department of Animal Health, Faculty of Veterinary Medicine, Complutense University of Madrid, Av. Puerta de Hierro s/n, 28040 Madrid, Spain

**Keywords:** Plasma, electrophoretogram, Chondrichthyan, Capillary zone electrophoresis, Catshark, Globulin, Aquarium research, Sebia

## Abstract

**Background:**

This study determined plasma protein electrophoresis (PPE) reference intervals in two elasmobranch species: the undulate skate (*Raja undulata*) and the nursehound shark (*Scyliorhinus stellaris*), using a reference population of 48 undulate skates (27 males, 21 females) and 62 nursehounds (32 males, 30 females), considered to be clinically healthy. Plasma samples were analyzed using capillary zone electrophoresis (CZE).

**Results:**

The undulate skate electrophoretogram resembled those previously reported in other batoids and could be divided into seven consistent fractions. No statistically significant differences were detected between sexes and developmental stages. The nursehound electrophoretogram was similar to that previously described in other shark species and could be divided into eight consistent fractions. Fraction 5% was significantly higher in juvenile nursehounds when compared to adults, while fraction 6 concentration and percentage were significantly higher in adults. Fraction 4% was higher in males than in females. Albumin band was not detected, and pre-albumin was negligible in both studied species. Alpha-globulins were predominant in the undulate skate, while beta-globulins were predominant in nursehounds. Statistically significant differences were found in all electrophoretogram fraction percentages and concentrations between the two species.

**Conclusion:**

To the authors knowledge, this is the first study reporting PPE values in undulate skates and nursehounds, and the first study using CZE in elasmobranch plasma. These findings can serve as a primary reference for health monitoring in both species and will add to the limited data available on PPE in elasmobranchs.

## Background

Rising standards and expectations for high-quality aquatic animal husbandry and veterinary medical care have required more professionals to become involved in the husbandry and clinical management of elasmobranchs [1]. This has induced an improvement in veterinary standards for sharks, rays, skates, and sawfish, supported by the advancement in modern veterinary medicine and technology, which increased our capacity to understand and monitor elasmobranch health [2, 3]. Preventive health management programs are increasingly being implemented in elasmobranchs maintained in public aquaria, and population health studies in free-ranging elasmobranch populations are also being progressively performed [4–8]. As part of these programs, routine examinations including detailed physical exams and blood analyses are rising in importance [4]. Although studies determining baseline reference intervals for common hematology and plasma biochemistry analytes are increasing both in free-ranging and aquarium-maintained elasmobranchs, baseline plasma protein electrophoresis (PPE) values have only been determined in very few elasmobranch species [3–5, 9]. In addition, due to the challenges associated to working with sharks and rays, previous studies determining PPE reference intervals (RI) in these species were limited by relatively low sample sizes (frequently under 30–40 individuals). These previous studies reported high interspecific variability however, thus illuminating the need for further studies to accurately evaluate variations in electrophoretogram fractions [3, 4, 9].

If possible, PPE analysis should be included in the routine health evaluation of elasmobranchs, as disease diagnosis in this group of animals can be challenging and the clinical application of PPE for health evaluation and disease diagnosis is useful, not only in mammal and avian species, but also in elasmobranchs [3, 9–11]. Variations in plasma protein values can indicate a subclinical disease before the animal shows clinical symptoms [4]. Moreover, together with the serum protein changes that can be seen in plasma chemistry analysis, PPE has been shown to be a beneficial testing method as it can be used to evaluate variations in plasma protein trends without the need for species-specific reagents and can more accurately quantitate lipoproteins, providing valuable information for assessing ongoing inflammatory processes [11, 12].

Due to the currently limited information on PPE in elasmobranchs, the goal of our study was to describe and determine RI for plasma protein electrophoretograms in two elasmobranch species frequently maintained under human care in Europe: the undulate skate (*Raja undulata*) and the nursehound shark (*Scyliorhinus stellaris*). Both species are medium-sized, oviparous, and benthic, with a similar geographical distribution (Northeast Atlantic continental shelf and the Mediterranean Sea) [13, 14]. Despite both species are frequently maintained in European aquaria due to their easy husbandry and handling compared to other elasmobranch species, studies involving these species are still relatively limited. To our knowledge, baseline PPE reference values have not yet been published for any skate and Scyliorhinid shark species. This study aimed to evaluate interspecific differences, as well as intraspecific differences in electrophoretogram values which could be related to differences in sex and developmental stage.

## Methods

### Animals

This study used heparinized blood plasma samples obtained during group veterinary health exams performed in 49 undulate skates (28 males and 21 females, 41 subadults and 8 adults) maintained at Oceanogràfic Aquarium of Valencia (Carrer d’Eduardo Primo Yúfera, 1B, Valencia 46,013, Spain; www.oceanografic.org) as well as 64 nursehounds (32 males and 32 females, 23 subadults and 41 adults) maintained in five different Spanish aquaria: Oceanogràfic Aquarium of Valencia, Finisterrae Aquarium (P.º Marítimo Alcalde Francisco Vázquez, 34, A Coruña 15,002, Spain; www.coruna.gal/mc2/es/aquarium-finisterrae), Bioparc Gijón Aquarium (Playa De Poniente, S/N, Gijón 33,212, Spain; www.acuariogijon.es); Museo Marítimo del Cantábrico (Av. de Severiano Ballesteros, s/n, Santander 39,004, Spain; www.museosdecantabria.es/museo-maritimo), and Sevilla Aquarium (Muelle de las Delicias, s/n, Sevilla 41,012, Spain; www.acuariosevilla.es). Sharks and skates were classified as subadults or adults based on size, using previously published adult size ranges, and sex was determined based on the presence/absence of claspers [15–17]. Sample collection during veterinary evaluations in undulate skate and nursehound populations for the purpose of this study was approved by the Animal Care and Welfare Committee at Oceanogràfic of Valencia and the Generalitat Valenciana under the project reference OCE-22-19.

### Environmental conditions

All sampled animals were maintained in tanks equipped with life support systems which included sand filters, protein fractionator, de-gas tower, biological filtration, ultra-violet (UV) and/or ozone disinfection. Tanks were supplied with natural sea water collected from coastal waters of the Mediterranean Sea in Oceanogràfic Aquarium and from the Atlantic Ocean in the rest of aquaria, previously processed via sand filters and/or UV disinfection. Water temperature ranged from 17 to 19 ºC; salinity was kept between 34.5 and 37 g/l; pH ranged from 7.8 to 8.1; redox was maintained between 200 and 350 mV; ammonia, nitrite, and nitrate were kept below 0.01, 0.05, and 100 ppm, respectively. Ambient air temperature ranged from 16 to 24 ºC. All sharks were sampled during summer (August 2021) and all skates were sampled during winter (February 2021). Animals were fed once daily, four to six days per week, and their diet consisted of thawed pieces of hake (*Merluccius spp*.), squid (*Loligo spp*.), prawn (*Penaus spp*.), herring (*Clupea spp*.), mackerel (*Scomber spp*.), and mussel (*Mytilus spp.*).

### Sample collection and processing

For blood collection, sharks and skates were captured using a rubber net, carefully handled, and placed in a dorsal decubitus to induce tonic immobility [18]. A volume of one ml peripheral blood was collected for routine veterinary clinical examination via venipuncture of the caudal hemal arch, using a 23- to 25-gauge needle attached to a 1–2 ml syringe [19, 20]. A ventral approach of the hemal arch was used in skates and a lateral approach was used in nursehounds while animals were manually restrained in water, with their head and gills submerged while the caudal third was brought outside of the water during sampling to avoid salt water contamination of the samples After blood collection, animals were weighed using a crane scale (GRAM CR 150-S, Gram Pre-cision S.L., l’Hospitalet de Llobregat 08907, Spain) and measures were taken for total length (snout to tip of the tail; TL, in cm) using a flexible nylon tape measure.

Blood samples were immediately placed in lithium heparin tubes (AQUISEL® 1 ml 12 × 55 mm REF: 1,501,406) and maintained at 4 ºC until centrifugation. Heparin tubes were centrifuged within 60 min from collection at a centrifugation speed of 448 g (2000 rpm) for 6 min at room temperature (24 ºC) using a portable centrifuge (Digital angle centrifuge 2615/1 Nahita-Blue, 100 mm rotor radius; LABOQUIMIA, Lardero 26,140, Spain). Plasma was collected using a micropipette and transferred to 1.5 ml Eppendorf tubes, which were frozen and stored at -18 ºC until processing as previous studies have demonstrated that a single freeze-thaw cycle does not produce statistically significant changes in PPE fractions [21]. Shark and skate plasma samples were thawed and processed for protein capillary zone electrophoresis (CZE) analysis within two months from blood collection using a Microcapillary Minicap Protein 6 Flex-Piercing (Sebia® Ref. 1232 v.7.48, Sebia Hispania S.A., Barcelona 008028, Spain). Before introducing the samples for electrophoresis analysis, a 1:40 ratio dilution (10 µl plasma to 390 µl Buffer) was performed using Capillarys Urine Dialysis Buffer (Sebia® Ref. 2013, Sebia Hispania S.A., BarCelona 08028, Spain) following indications provided by the team of Sebia Hispania S.A., which was also previously diluted at 50:50 ratio using distilled water following manufacturer recommendations. Absolute fraction values (g/l) were determined by multiplying the percentages for each fraction by total protein concentrations measured using a Beckman Coulter® Chemistry Analyzer (A91961-AU480 Chemistry Analyzer, Beckman Coulter S.L.U., Alcobendas 28,108, Spain). The coefficient of variation (CV) was always under 8% or all fractions studied in each one of the studied species. Samples were selected to form part of the RI after the detailed evaluation of every animal’s medical history and the completion of physical examinations (which included a hematological and biochemical analysis) to evaluate the condition of each individual sampled. For this study, we divided the electrophoretogram into several fractions following the guidelines established in previous studies with elasmobranchs, which already defined five different fractions in ray and shark species [3, 4, 9, 22, 23]. Caution was taken to delete the injection peak and the end delimiter produced by the urine buffer before electrophoretogram interpretation and division into fractions for its analysis. As done in previous PPE studies with elasmobranchs, and in the absence of studies defining which proteins correspond to each electrophoretogram fraction, the classification of the different fractions as pre-albumin, alpha-, beta-, and gamma-globulins, was made based on their similarity in migration properties with respect to other taxa [11]. The further division of protein fractions migrating in the alpha- and beta-globulin regions into several subfractions was possible as the different peaks in the curve were consistent and reproducible among all the individuals sampled. Any abnormality in an animal’s medical history, as well as in the thorough external examination and bloodwork, excluded the affected individual from the reference population. All sharks and skates were fasted for a minimum period of 24 h before sampling to avoid possible imbalances in PPE results [24].

### Statistical analyses

Reference intervals were determined using the RefVal adv. 2.0 Excel add in and the MedCalc® statistical software [25]. Following the recommended guidelines published by the American Society of Veterinary Clinical Pathology, Dixon’s outlier range statistics and Tukey tests were used for the identification of outliers [26, 27]. If an outlier was detected in one electrophoretogram fraction, the entire data set from that animal was eliminated as all fraction percentages would be affected. Statistical analyses were then rerun for all samples. Total protein and plasma protein fractions were analysed for normality. Shapiro Wilk and Kolmogorov-Smirnov tests with Lilliefors corrections were used to determine Gaussian distribution. We established a *p*-value of 0.20 as a cut off value for Shapiro Wilk and a *p*-value of 0.26 as a cut off value for Kolmogorov-Smirnov tests instead of 0.05, following the previously established recommendations for RI determination using small sample sizes [28]. Non-parametric 90% confidence intervals (CI) were calculated using a bootstrap method. To evaluate differences between sexes (male vs. female) and developmental stage (adult vs. subadult) a non-parametric (Mann-Whitney U test) was used for non-normally distributed data, and a parametric test (Student’s t-test) was used for data with a normal distribution. Furthermore, differences between subgroups (adult female vs. adult male vs. subadult female vs. subadult male) were evaluated using a Kruskal-Wallis test and Dunn’s post hoc tests with Bonferroni corrections (for non-normally distributed data) or an ANOVA (for data showing a normal distribution); significance for these tests was set at *p* < 0.05. Statistical comparison between sexes in adult skates and comparison involving unhealthy individuals was not possible due to the small sample size. For interspecific comparison, due to the different number of consistent electrophoretogram fractions observed in undulate skates (seven fractions) and nursehounds (eight fractions), electrophoretograms were divided for both species into: pre-albumin, alpha-, beta-, and gamma-globulin fractions, wherein total alpha- and beta-globulin fractions were calculated as the sum of the corresponding alpha- and beta-sub-fractions. Once total alpha- and beta-globulin fractions were calculated, interspecific statistical comparison was performed using a Mann-Whitney U test. Alpha-2-/Beta-globulin ratio was calculated in undulate skate by dividing fraction 3 by the sum of the fractions 4, 5, and 6; and in nursehound sharks by dividing the sum of fractions 3 and 4 by the sum of fractions 5, 6, and 7. Statistical comparison for this ratio between species was performed using a Mann-Whitney U test. The statistical software package RStudio® (Version 1.2.504; Rstudio Team, 2020. Boston 02210, USA; ww.rstudio.com) and SPSS Statistics 25.9 (IBM Corp New York, USA; www.ibm.com) were used for statistical analyses.

## Results

Two adult female nursehounds were excluded from the study during the preliminary physical examination due to the presence of conspecific bite wounds. One adult male undulate skate was also excluded during the physical examination due to the presence of a laceration in the oral cavity which affected the lower jaw and evident alterations in hematological and plasma chemical values. Two juvenile skates which were apparently healthy upon physical examination, presented outliers in their electrophoretograms and were eliminated from the reference population. The remaining 62 sharks (32 males and 30 females, 23 subadults and 39 adults) and 48 skates (27 males and 21 females, 41 subadults and 7 adults) were considered clinically healthy, and their plasma samples were analyzed for RI determination. All animals showed a fast recovery after sampling and no clinical signs were detected in any of the animals one month after sampling. Median (min-max range) body weight was 2.43 (0.7–7.4) kg in nursehounds and 1.3 (0.1–4.8) kg in undulate skates. Median (range) total length was 76.0 (51.0-113.0) cm in nursehounds and 51.3 (31.5–81.0) cm in skates.

The undulate skate electrophoretogram resembled those previously reported in other batoids, such as the cownose ray (*Rhinoptera bonasus*) and could be divided into seven consistent fractions (migrating in regions considered equivalent to pre-albumin, alpha-1, alpha-2, beta-1, beta-2, beta-3, and gamma globulins) [3]. Descriptive statistics for PPE parameters in the undulate skate are shown in Table 1. A comparison for electrophoretogram values in undulate skates between sexes is provided in Table 2, and a comparison between developmental stages is provided in Table 3. No statistically significant differences were detected between sexes (males vs. females), developmental stages (adults vs. subadults) (*p* > 0.05; Mann-Whitney U test), nor between subgroups (adult female vs. subadult female vs. adult male vs. subadult male) (*p* > 0.05; Dunn’s test, Bonferroni-adjusted).

**Table 1:** Reference intervals for plasma protein electrophoresis fractions in the undulate skate (*Raja undulata*), using the Sebia Minicap Protein 6 Flex-Piercing.

Analyte (unit)	n	Mean	SD	Median	Min	Max	RI	LRL	URL	Distr	Method	P
TP (g/l)	48	29.33	3.19	29.00	22.00	36.00	22.23–35.78	22.00-24.45	34.78-36.00	G	P	0.254
Fraction 1 (%)	48	0.45	0.24	0.40	0.10	1.00	0.12–0.98	0.10–0.20	0.90-1.00	NG	NP	< 0.01
Fraction 2 (%)	48	12.98	5.10	12.75	4.40	24.20	4.47–23.93	4.40–5.14	20.74–24.20	G	P	0.423
Fraction 3 (%)	48	42.65	3.17	43.15	36.10	48.90	36.21–48.68	36.10-36.97	46.70–48.90	G	P	0.408
Fraction 4 (%)	48	33.36	3.78	32.40	24.30	42.30	24.84–42.05	24.30–28.40	38.90–42.30	G	P	0.879
Fraction 5 (%)	48	4.84	1.54	4.60	2.30	8.40	2.30–8.40	2.30–2.86	7.83–8.40	NG	NP	< 0.01
Fraction 6 (%)	48	3.88	1.54	3.50	1.40	8.30	1.51–8.17	1.40-2.00	7.03–8.30	NG	NP	0.031
Fraction 7 (%)	48	1.83	0.55	1.95	0.80	3.10	0.82–3.10	0.80–0.92	2.50–3.10	G	P	0.222
Fraction 1 (g/l)	48	0.13	0.07	0.11	0.03	0.32	0.04–0.31	0.03–0.05	0.26–0.32	NG	NP	< 0.01
Fraction 2 (g/l)	48	3.77	1.47	3.70	1.20	7.99	1.28–7.59	1.20–1.64	5.83–7.99	G	P	0.358
Fraction 3 (g/l)	48	12.54	1.92	12.30	8.62	17.24	8.92–16.99	8.62–10.04	16.02–17.24	NG	NP	0.149
Fraction 4 (g/l)	48	9.80	1.68	9.61	5.83	14.81	6.13–14.58	5.83–7.90	12.95–14.81	NG	NP	0.011
Fraction 5 (g/l)	48	1.40	0.43	1.30	0.69	2.44	0.72–2.44	0.69–0.87	2.32–2.44	NG	NP	< 0.01
Fraction 6 (g/l)	48	1.14	0.49	1.00	0.39	2.74	0.43–2.68	0.39–0.62	1.95–2.74	NG	NP	< 0.01
Fraction 7 (g/l)	48	0.54	0.18	0.55	0.22	0.90	0.23–0.90	0.22–0.26	0.85–0.90	NG	NP	0.199
Alpha-2-/Beta-globulin ratio	48	1.03	0.12	1–01	0.82	1.33	0.82–1.32	0.82–0.88	1.24-1.33	NG	NP	0.173

n, number of individuals; SD, standard deviation; RI, reference intervals; LRL, 90% confidence interval of the lower reference limit; URL, 90% confidence interval of the upper reference limit (90% confidence intervals of the limits determined nonparametrically used a bootstrap method); Distr, distribution; P, *p*-value calculated using Saphiro-Wilk test (cut off value was set at 0.20); G, Gaussian; NG, non-Gaussian; P, parametric; NP, non-parametric TP, total proteins in plasma

**Table 2:** Plasma protein electrophoresis comparison between female and male undulate skates (*Raja undulata*) maintained under human care. No statistically significant differences were detected between sexes (p *>* 0.05; Mann-Whitney U test or *Student’s t-test)

	Undulate skate (*Raja undulata*)										
	**Female (n = 21)**					**Male (n = 27)**					
**Analyte (unit)**	**Mean**	**SD**	**Median**	**Min**	**Max**	**Mean**	**SD**	**Median**	**Min**	**Max**	**P**
TP (g/l)	29.62	3.37	30.00	22.00	35.00	29.11	3.09	28.00	24.00	36.00	0.589*
Fraction 1 (%)	0.49	0.23	0.40	0.20	0.90	0.43	0.24	0.40	0.10	1.00	0.461
Fraction 2 (%)	12.63	4.70	11.40	5.20	21.30	13.61	5.34	13.40	4.40	24.20	0.338*
Fraction 3 (%)	43.03	2.85	43.30	37.20	47.20	42.37	3.43	42.10	36.10	48.90	0.483*
Fraction 4 (%)	32.80	3.43	32.10	26.70	38.90	33.65	4.04	33.70	24.30	42.30	0.550*
Fraction 5 (%)	4.97	1.76	4.60	2.30	8.40	4.53	1.21	4.50	2.30	7.60	0.228
Fraction 6 (%)	4.30	1.57	3.90	2.10	8.30	3.57	1.46	3.40	1.40	7.30	0.100
Fraction 7 (%)	1.79	0.43	1.90	0.90	2.40	1.84	0.64	1.90	0.80	3.10	0.917*
Fraction 1 (g/l)	0.15	0.08	0.13	0.05	0.32	0.13	0.07	0.11	0.03	0.26	0.448
Fraction 2 (g/l)	3.71	1.35	3.58	1.64	5.88	0.39	1.55	3.75	1.20	0.80	0.435*
Fraction 3 (g/l)	12.79	2.01	12.90	8.62	16.07	1.35	1.87	11.89	9.94	17.24	0.313
Fraction 4 (g/l)	9.69	1.34	9.60	7.31	12.75	9.85	1.92	9.60	5.83	14.81	0.987
Fraction 5 (g/l)	1.45	0.49	1.34	0.69	2.44	1.31	0.33	1.23	0.81	2.13	0.101
Fraction 6 (g/l)	1.30	0.58	1.09	0.63	2.74	1.03	0.38	0.96	0.39	1.82	0.146
Fraction 7 (g/l)	0.53	0.15	0.58	0.24	0.72	0.54	0.20	0.53	0.22	0.90	0.519

n, number of individuals; SD, standard deviation; TP, total proteins in plasma

**Table 3:** Plasma protein electrophoresis comparison between adult and subadult undulate skates (*Raja undulata*) maintained under human care. No statistically significant differences were detected between sexes (*p >* 0.05; Mann-Whitney U test or *Student’s t-test)

	Undulate skate (*Raja undulata*)										
	**Adult (n = 7)**					**Subadult (n = 41)**					
**Analyte (unit)**	**Mean**	**SD**	**Median**	**Min**	**Max**	**Mean**	**SD**	**Median**	**Min**	**Max**	**P**
TP(g/l)	28.71	1.89	28.00	26.00	32.00	29.44	3.37	29.00	22.00	36.00	0.584*
Fraction 1 (%)	0.40	0.15	0.40	0.20	0.60	0.46	0.25	0.40	0.10	1.00	0.722
Fraction 2 (%)	12.16	2.64	11.20	9.20	17.20	13.12	5.42	12.8	4.40	24.2	0.649*
Fraction 3 (%)	41.97	3.13	42.80	36.10	44.80	42.77	3.21	43.20	36.60	48.9	0.544*
Fraction 4 (%)	33.40	2.77	33.90	28.90	36.10	33.35	3.96	32.30	24.30	42.3	0.974*
Fraction 5 (%)	5.53	1.50	6.20	3.50	7.60	4.72	1.53	4.60	2.30	8.40	0.219
Fraction 6 (%)	4.64	1.42	4.60	1.90	6.10	3.75	1.54	3.50	1.40	8.30	0.057
Fraction 7 (%)	1.90	0.80	2.10	0.90	3.10	1.82	0.50	1.90	0.80	3.10	0.731*
Fraction 1 (g/l)	0.12	0.05	0.11	0.05	0.19	0.14	0.08	0.12	0.03	0.30	0.650
Fraction 2 (g/l)	3.49	0.75	3.58	2.58	4.82	3.81	1.56	3.72	1.20	8.00	0.594*
Fraction 3 (g/l)	12.07	1.46	11.98	10.11	14.34	12.63	1.99	12.42	8.62	17.2	0.526
Fraction 4 (g/l)	9.57	0.76	9.75	8.09	10.44	9.84	1.80	9.60	5.83	14.8	0.988
Fraction 5 (g/l)	1.58	0.42	1.61	0.98	2.13	1.37	0.43	1.29	0.69	2.40	0.225
Fraction 6 (g/l)	1.34	0.44	1.29	0.55	1.95	1.11	0.50	0.95	0.39	2.70	0.082
Fraction 7 (g/l)	0.55	0.23	0.62	0.25	0.87	0.54	0.17	0.54	0.22	0.90	0.770

n, number of individuals; SD, standard deviation; TP, total proteins in plasma

The nursehound electrophoretogram was similar to that previously described in other shark species and could be divided into eight consistent fractions (migrating in the regions and considered equivalent to pre-albumin, alpha-1, alpha-2.1, alpha-2.2, beta-1, beta-2, beta-3, beta-4, and gamma globulins). Descriptive statistics for PPE parameters in the nursehound are presented in Table 4. Fraction 5% was significantly higher in juvenile nursehounds (median 30.70%) when compared to adults (median 29.03%) (*p* = 0.038; Mann-Whitney U test), while fraction 6 concentration and percentage was significantly higher in adults (3.55 g/l* and 14.32%) when compared to juveniles (2.87 g/l* and 10.91%) (*p* < 0.01; Mann-Whitney U test or *Student’s t-test). Fraction 8 concentration and percentage were also significantly higher in adult nursehounds (median 1.48 g/l and 6.38%) compared to juveniles (median 1.05 g/l and 4.65%) (p < 0.01; Student’s t-test). Statistically significant differences were also detected between sexes, as fraction 4% was higher in males (median 27.70%) than in females (median 26.85%) (*p* = 0.030; Mann-Whitney U test), with no statistically significant differences between subgroups (*p* > 0.05; Dunn’s test, Bonferroni-adjusted). A comparison for electrophoretogram fraction values between sexes is provided in Table 5, and a comparison between developmental stages in provided in Table 6.

**Table 4:** Reference intervals for plasma protein electrophoresis fractions in nursehounds (*Scyliorhinus stellaris*), using the Sebia Minicap Protein (E) 6 Flex-Piercing.

Analyte (unit)	n	Mean	SD	Median	Min	Max	RI	LRL	URL	Distr	Method	P
TP (g/l)	62	24.66	4.22	23.50	19.00	36.00	19.58–34.28	19.00–20.00	32.00–36.00	NG	NP	< 0.01
Fraction 1 (%)	62	0.74	0.35	0.64	0.21	1.50	0.30–1.49	0.22–0.31	1.43–1.50	NG	NP	< 0.01
Fraction 2 (%)	62	1.98	0.73	1.81	0.80	3.90	0.82–3.73	0.80–1.09	3.27–3.90	NG	NP	0.017
Fraction 3 (%)	62	9.99	1.88	10.15	5.60	14.50	6.19–13.79	5.51–6.85	13.13–14.53	NG	NP	0.200
Fraction 4 (%)	62	26.62	3.46	27.16	16.60	33.68	17.61–33.58	16.60–20.40	31.39–33.68	NG	NP	0.011
Fraction 5 (%)	62	28.79	3.86	29.72	19.80	34.99	19.97–34.80	19.80-22.68	34.04–34.99	NG	NP	< 0.01
Fraction 6 (%)	62	13.22	3.16	12.69	7.68	19.66	8.06–19.65	7.68–9.21	19.21–19.66	NG	NP	0.038
Fraction 7 (%)	62	13.25	4.32	12.97	5.56	24.80	5.56–24.05	5.56–6.87	21.44–24.80	NG	NP	0.200
Fraction 8 (%)	62	5.40	1.59	5.40	1.80	8.10	2.19–8.64	1.66–2.79	8.06–9.24	NG	NP	0.200
Fraction 1 (g/l)	62	0.18	0.09	0.17	0.04	0.42	0.05–0.40	0.05–0.07	0.32–0.42	NG	NP	0.071
Fraction 2 (g/l)	62	0.49	0.21	0.43	0.19	1.17	0.22–1.03	0.19–0.25	0.89–1.17	NG	NP	0.014
Fraction 3 (g/l)	62	2.46	0.63	2.23	1.13	4.06	1.19–3.74	0.96–1.41	3.50–3.99	NG	NP	0.200
Fraction 4 (g/l)	62	6.59	1.55	6.10	4.14	10.06	4.27–9.88	4.14–4.79	9.49–10.06	NG	NP	< 0.01
Fraction 5 (g/l)	62	7.12	1.68	6.77	4.67	11.09	4.67–10.94	4.67–4.86	10.19–11.09	NG	NP	0.037
Fraction 6 (g/l)	62	3.25	0.90	3.10	1.69	5.75	1.44–5.05	1.11–1.76	4.71–5.38	NG	NP	0.200
Fraction 7 (g/l)	62	3.22	1.07	3.09	1.22	6.82	1.31–6.32	1.22–1.85	5.05–6.82	NG	NP	0.200
Fraction 8 (g/l)	62	1.35	0.51	1.29	0.52	2.57	0.32–2.37	0.14–0.52	2.17–2.55	NG	NP	0.182
Alpha-2-/ Beta-globulin ratio	62	0.67	0.12	0.67	0.35	1.06	0.50–1.09	0.50–0.52	0.89–1.29	NG	NP	< 0.01

n, number of individuals; SD, standard deviation; RI, reference intervals; LRL, 90% confidence interval of the lower reference limit; URL, 90% confidence interval of the upper reference limit (90% confidence intervals of the limits determined nonparametrically used a bootstrap method); Distr, distribution; P, p-value determined using a Kolmogorov-Smirnov normality test with Lilliefors corrections (cut off value was set at 0.26); G, Gaussian; NG, non-Gaussian; P, parametric; NP, non-parametric TP, total proteins in plasma.

**Table 5:** Plasma protein electrophoresis comparison between female and male nursehounds (*Scyliorhinus stellaris*) maintained under human care. Bold font indicates statistically significant differences between sexes (*p* < 0.05; Mann-Whitney U test)

				Nursehound (*Scyliorhinus stellaris*)										
						**Female (n = 30)**					**Male (n = 32)**			
**Analyte (unit)**	**Mean**	**SD**	**Median**		**Min**	**Max**	**Mean**	**SD**	**Median**	**Min**		**Max**	**P**	
TP (g/l)	25.03	4.58	24.00		20.00	36.00	24.31	3.90	23.00	19.00		32.00	0.640	
Fraction 1 (%)	0.81	0.37	0.74		0.31	1.50	0.68	0.33	0.63	0.22		1.48	0.265	
Fraction 2 (%)	2.06	0.67	2.00		0.84	3.30	1.91	0.78	1.76	0.80		3.90	0.260	
Fraction 3 (%)	9.85	1.90	10.55		6.00	12.70	10.12	1.89	10.14	5.60		14.50	0.944	
**Fraction 4 (%)**	**25.48**	**3.85**	**26.85**		**16.60**	**33.68**	**27.70**	**2.68**	**27.58**	**21.66**		**33.50**	**0.030**	
Fraction 5 (%)	28.33	4.51	28.80		19.80	34.99	29.22	3.15	29.76	20.70		34.66	0.693	
Fraction 6 (%)	13.71	3.29	12.90		8.99	19.64	12.77	3.00	12.32	7.68		19.66	0.278	
Fraction 7 (%)	14.18	4.68	13.29		5.56	24.80	12.36	3.82	12.50	5.56		20.80	0.195	
Fraction 8 (%)	5.58	1.50	5.41		2.30	8.10	5.23	1.68	5.35	1.80		7.72	0.463	
Fraction 1 (g/l)	0.20	0.09	0.18		0.07	0.43	0.17	0.09	0.15	0.05		0.33	0.172	
Fraction 2 (g/l)	0.52	0.22	0.45		0.26	1.17	0.47	0.21	0.41	0.19		0.94	0.254	
Fraction 3 (g/l)	2.47	0.67	2.35		1.38	4.06	2.46	0.61	2.35	1.34		3.98	0.977	
Fraction 4 (g/l)	6.39	1.65	5.96		4.14	10.06	6.77	1.46	6.59	4.77		9.57	0.237	
Fraction 5 (g/l)	7.14	1.91	6.96		4.67	10.83	7.11	1.46	6.65	4.97		11.09	0.921	
Fraction 6 (g/l)	3.40	0.93	3.13		2.16	5.75	3.10	0.85	3.06	1.69		5.38	0.291	
Fraction 7 (g/l)	3.49	1.18	3.21		1.80	6.81	2.96	0.89	2.82	1.22		4.99	0.078	
Fraction 8 (g/l)	1.42	0.54	1.29		0.52	2.71	1.27	0.46	1.31	0.52		2.01	0.352	

n, number of individuals; SD, standard deviation; TP, total proteins in plasma

**Table 6:** Plasma protein electrophoresis comparison between adult and subadult nursehounds (*Scyliorhinus stellaris*) maintained under human care. Bold font indicates statistically significant differences between sexes (*p* < 0.05; Mann-Whitney U test)

	Nursehound (*Scyliorhinus stellaris*)										
			**Adult (n = 39)**					**Juvenile (n = 23)**			
**Analyte (unit)**	**Mean**	**SD**	**Median**	**Min**	**Max**	**Mean**	**SD**	**Median**	**Min**	**Max**	**P**
TP (g/l)	24.92	4.46	24.00	19.00	36.00	24.22	3.84	23.00	20.00	3.30	0.781
Fraction 1 (%)	0.70	0.34	0.62	0.22	1.49	0.81	0.37	0.74	0.31	1.50	0.294
Fraction 2 (%)	2.04	0.71	1.81	0.80	3.60	1.89	0.76	1.78	0.84	3.90	0.332
Fraction 3 (%)	9.71	1.90	10.15	5.60	14.00	10.47	1.81	10.15	6.77	14.50	0.182
Fraction 4 (%)	26.19	3.34	26.96	16.60	31.20	27.36	3.61	27.30	18.70	33.68	0.315
**Fraction 5 (%)**	**28.07**	**3.77**	**29.03**	**19.80**	**34.34**	**30.00**	**3.79**	**30.70**	**23.10**	**34.99**	**0.038**
**Fraction 6 (%)**	**14.32**	**2.91**	**14.38**	**9.60**	**19.66**	**11.37**	**2.70**	**10.91**	**7.68**	**19.20**	**< 0.01**
Fraction 7 (%)	13.12	5.00	13.49	5.56	24.80	13.46	2.94	12.61	9.97	22.30	0.754
**Fraction 8 (%)**	**5.85**	**1.54**	**6.28**	**1.80**	**8.10**	**4.64**	**1.41**	**4.65**	**2.30**	**7.70**	**< 0.01**
Fraction 1 (g/l)	0.18	0.09	0.17	0.05	0.36	0.20	0.09	0.18	0.07	0.43	0.461
Fraction 2 (g/l)	0.52	0.23	0.49	0.19	1.17	0.45	0.19	0.41	0.24	0.94	0.308
Fraction 3 (g/l)	2.43	0.67	2.31	1.34	4.06	2.53	0.57	2.57	1.69	3.98	0.493
Fraction 4 (g/l)	6.55	1.60	6.18	4.37	10.06	6.65	1.50	6.02	4.14	9.65	0.560
Fraction 5 (g/l)	7.01	1.66	6.66	4.67	10.33	7.32	1.74	6.97	4.86	11.09	0.450
**Fraction 6 (g/l)**	**3.55**	**0.91**	**3.40**	**1.92**	**5.75**	**2.72**	**0.59**	**2.87**	**1.69**	**4.03**	**< 0.01**
Fraction 7 (g/l)	3.21	1.25	2.99	1.22	6.82	3.23	0.67	3.14	2.19	4.46	0.475
**Fraction 8 (g/l)**	**1.48**	**0.53**	**1.57**	**0.52**	**2.57**	**1.12**	**0.39**	**1.05**	**0.55**	**2.00**	**< 0.01**

n, number of individuals; SD, standard deviation; TP, total proteins in plasma

**Fig. 1:**
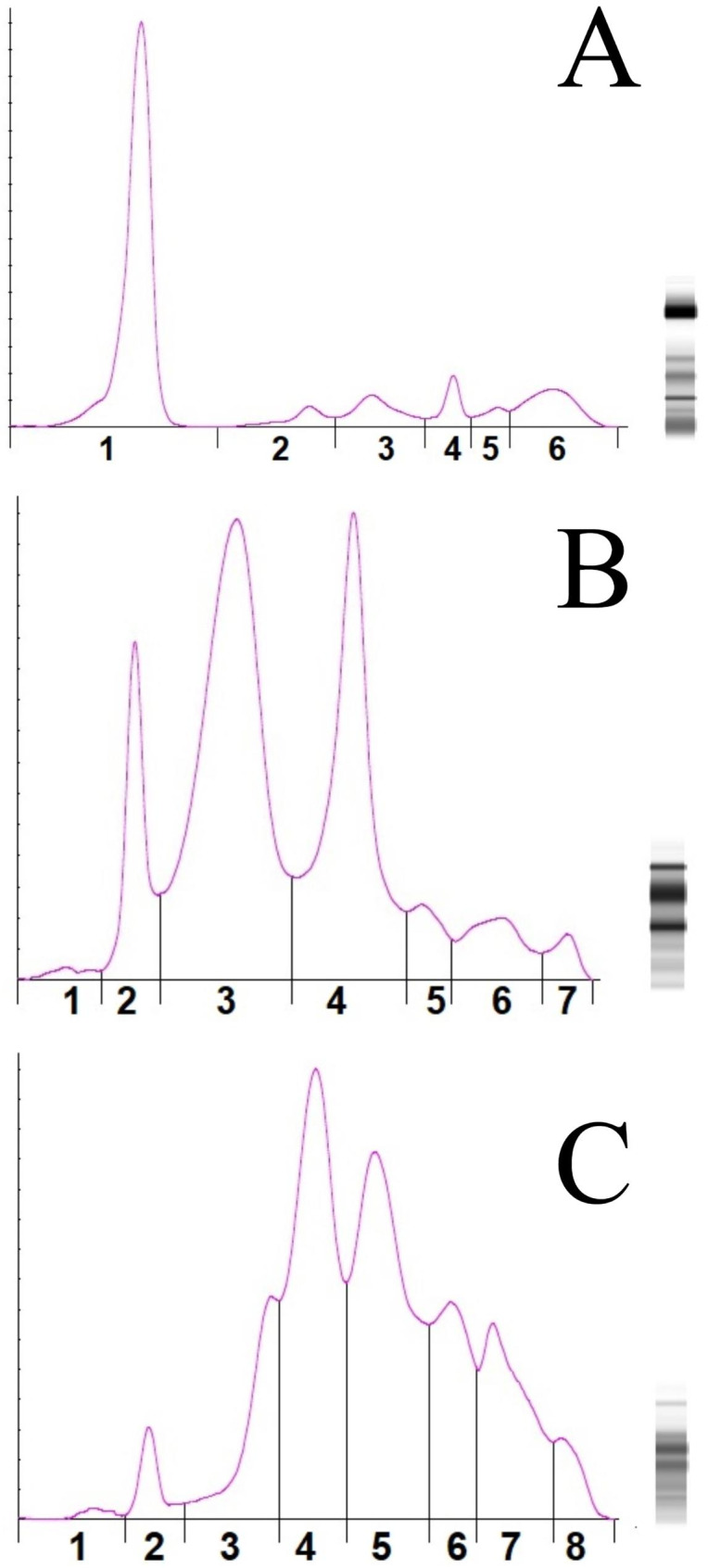
(**A**) Representative plasma protein electrophoretogram of a human control. Six consistent fractions are identified. Fractions one to six are equivalent to albumin, alpha-1, alpha 2, beta-1, beta-2, and gamma globulins. (**B**) Representative plasma protein electrophoretogram of a clinically healthy undulate skate (*Raja undulata*). Seven consistent fractions are identified in the species. Fractions one to seven are equivalent to pre-albumin, alpha-1, alpha 2, beta-1, beta-2, beta-3, and gamma globulins. (**C**) Representative plasma protein electrophoretogram of a clinically healthy nursehound (*Scyliorhinus stellaris*). Eight consistent fractions are identified in the species. Fractions one to eight are equivalent to pre-albumin, alpha-1, alpha-2.1, alpha 2.2, beta-1, beta-2, beta-3, and gamma globulins. The bands on the right of each electrophoretogram correspond to a digital gel electrophoresis representation for plasma each sample.

As previously reported in PPE studies with elasmobranchs, albumin band was not detected in undulate skates and in nursehounds, and proteins migrating in the pre-albumin region were negligible in both species [3, 9]. Alpha-globulins were predominant in the undulate skate, while beta-globulins were predominant in nursehounds. Gamma-globulin fraction was easily identified in both species, with values close to those previously reported in other elasmobranch species. Statistically significant differences were found in all electrophoretogram fraction percentages and concentrations between undulate skates and nursehounds (*p* < 0.05; Mann-Whitney U test). A representative PPE profile from a clinically healthy undulate skate and a clinically healthy nursehound is presented in Fig. 1. A comparison between the two studied elasmobranch species is provided in Table 7

**Table 7:** Plasma protein electrophoresis comparison between the undulate skate (Raja undulata) and the nursehound (*Scyliorhinus stellaris*) maintained under human care. Distribution was non-Gaussian for all represented parameters (p < 0.05; Kolmogorov-Smirnov normality test). Statistically significant differences were detected between species for all the studied parameters (*p* < 0.01; Mann-Whitney U test)

	Undulate skate (*Raja undulata*) (n = 48)					Nursehound (*Scyliorhinus stellaris*) (n = 62)					
**Analyte (unit)**	**Mean**	**SD**	**Median**	**Min**	**Max**	**Mean**	**SD**	**Median**	**Min**	**Max**	**P**
TP (g/l)	29.33	3.19	29.00	22.00	36.00	24.66	4.22	23.50	19.00	36.00	**< 0.01**
Pre-albumin (%)	0.44	0.24	0.40	0.10	1.00	0.74	0.35	0.64	0.22	1.50	**< 0.01**
Alpha-globulin (%)	55.85	3.79	56.40	46.90	61.90	38.60	4.31	38.59	25.20	51.90	**< 0.01**
Beta-globulin (%)	41.89	3.73	41.30	35.80	50.80	55.26	4.02	55.30	45.20	67.90	**< 0.01**
Gamma-globulin (%)	1.82	0.55	1.90	0.80	3.10	5.40	1.59	5.40	1.80	8.10	**< 0.01**
Pre-albumin (g/l)	0.13	0.07	0.11	0.03	0.32	0.18	0.09	0.17	0.05	0.43	**< 0.01**
Alpha-globulin (g/l)	16.37	2.02	16.16	11.77	20.27	9.55	2.12	8.91	6.60	14.67	**< 0.01**
Beta-globulin (g/l)	12.29	1.79	12.19	8.83	16.52	13.59	2.28	13.16	10.49	19.69	**< 0.01**
Gamma-globulin (g/l)	0.53	0.18	0.54	0.22	0.90	1.35	0.51	1.29	0.52	2.57	**< 0.01**

n, number of individuals; SD, standard deviation; TP, total proteins in plasma. Please note: Pre-albumin was considered fraction 1 in undulate skates and nursehounds. To allow interspecific comparison, alpha-globulin fraction was calculated as the sum of fractions 2 and 3 (equivalent to alpha-1 and alpha-2-globulins) in undulate skates, and fractions 2, 3 and 4 (equivalent to al-pha-1, alpha-2.1 and alpha-2.2 globulins) in nursehounds. Beta-globulin fraction was calculated as the sum of fractions 4, 5 and 6 (equivalent to beta-1, beta-2, and beta-3-globulins) in undulate skates, and fractions 5, 6 and 7 (equivalent to beta-1, beta-2, and beta-3-globulin fractions) in nursehounds. Gamma-globulin fraction represented fraction 7 in skates and fraction 8 in nursehounds.

## Discussion

To the authors knowledge, this is the first study evaluating PPE in elasmobranchs using CZE. Previous studies with elasmobranchs used Agarose Gel Electrophoresis (AGE), which is still the most prevalent method used in veterinary settings. However, CZE, due to its higher accuracy and easiness of use through automatization, is increasingly replacing AGE as a research and diagnostic tool for PPE analysis in many species including marine animals [29]. CZE has proven to consistently resolve electrophoretogram protein fractions that are frequently not differentiated by using AGE [30]. Previous studies evaluating PPE in sharks and rays divided these species’ electrophoretograms into five fractions equivalent to pre-albumin, albumin, alpha-, beta-, and gamma-globulins. Our study was able to classify the electrophoretograms into seven consistent fractions in the undulate skate and eight in the nursehound shark, which was consistent across all the animals sampled [3–5, 9, 31]. This division of the fractions into smaller ones was based on previous findings from cownose rays [3]. In this study, CZE allowed the classification of the proteins migrating in the alpha- and beta-globulin regions into several sub-fractions which were consistent throughout the sampled population of the study. Although further studies are warranted to completely understand the composition and clinical implications of the different sub-fractions, the possibility of dividing the electrophoretogram into several small consistent fractions will allow for more accurate interpretation of the variations occurring in the electrophoretogram [21, 29, 30].

In this study, significant interspecific variations were found for plasma total proteins between the two studied species: median 29.00 g/l in undulate skates and 23.50 g/l in nursehounds (*p* < 0.01; Mann-Whitney U test). Significant variations in plasma total protein values have been previously described between different elasmobranch species, with median 23.00 g/l in the Atlantic sharpnose shark (*Rhizoprionodon terraenovae*), 28.00 g/l in the bonnethead shark (*Sphyrna tiburo*), and 21.00 g/l in the spiny dogfish (*Squalus acanthias*) [5].

Our PPE study revealed low quantities of proteins migrating in the pre-albumin region of the electrophoretogram for undulate skates (median 0.44% and 0.13 g/l) and nursehounds (median 0.64% and 0.17 g/l), which may contain high-density proteins as described in other elasmobranchs, and which we characterized as “Fraction 1” following the classification established in previous PPE studies with elasmobranchs in an effort to standardize electrophoretogram fractions for these species [3, 22]. Low percentages and concentrations for fraction 1 agree with previous PPE studies with elasmobranchs which also reported similar values for this fraction: 1.30% and0.30 g/l in the Atlantic sharpnose shark, 0.71% and0.20 g/l in the bonnethead shark [5].

In this study, a lipoprotein fraction migrating in the albumin region was not detected in undulate skates or nursehounds. The presence of albumin in elasmobranchs is currently being questioned, with previous PPE studies reporting negligible protein percentages and concentrations in the albumin band in bamboo sharks (*Chiloscyllium Plagiosu*m) and bonnethead sharks [5, 9]. Furthermore, a study examining plasma samples from eight Chondrichthyan species using agarose gel electrophoresis (AGE) and cellulose acetate electrophoresis found albumin to be absent in all species studied, including sharks and skates, and suggested that most elasmobranch species do not have detectable levels of non-esterified fatty acids (and the respective binding proteins), binding proteins to long-chain fatty acids instead [32].

Plasma proteins migrating in the alpha-globulin region formed two consistent electrophoretogram fractions (fraction 2 and 3; named alpha-1 and alpha-2 globulin fractions) in the undulate skate, and three consistent fractions (fractions 2, 3 and 4; named alpha-1, alpha-2.1 and alpha-2.2 globulin fractions) in the nursehound shark. Proteins migrating in this region in different avian, reptile, and mammal species, have been characterized as alpha-2 macroglobulin, hapto-globulin, and ceruloplasmin; though the composition of these fractions in elasmobranchs has not been yet determined [4, 22]. As previously described in other elasmobranchs species such as the blacknose shark (*Carcharhinus acronotus*), the blacktip shark (*Carcharhinus limbatus*), the bull shark (*Carcharhinus leucas*), the lemon shark (*Negaprion brevirostris*), the sandbar shark (*Carcharhinus plumbeus*), the tiger shark (*Galeocerdo cuvier*), the cownose ray, the bamboo shark and the bonnethead shark, alpha-1 fraction was smaller than alpha-2 fraction also in undulate skates and nursehounds [3, 4, 9, 23]. Fraction 2 (equivalent to alpha-1 globulin) was significantly higher in undulate skates (median 12.75%, 1.47 g/l) compred to nursehounds (median 1.81% and 0.43 g/l), s both values between those reported for this fraction in other elasmobranch species such as the sand tiger shark (0.28% and 0.13 g/l), amboo shark (median 1.42% and 1.00 g/l) o bonnethead shark (4.82%, 2.80 g/l) showgreat interspecific variations. Proteins migrating in the alpha-2 region could be divided into two well-defined and preserved subfractions in nursehounds (equivalent to alpha 2 − 1 and 2–2 globulin fractions) while this subdivision was not observed in undulate skates. In addition, fraction 3 (equivalent to alpha-2 globulin) was also higher in undulate skates than in nursehounds (median 43.15% and 12.30 g/l) ompared to the sum of fractions 3 (alpha-2.1 globulins) and 4 (alpha-2.2 globulins) (median 37.31% and 8.33 g/l). ignificant interspecific variations have been described in alpha-2 globulins between the different elasmobranchs, with median values ranging from 12.0% to 7.2 g/l in ownose rays, 26.31% and 15.0 g/l inthe sand tiger shark, and 36.22% and 22.80 g/l i the bonnethead shark [3–5, 31].

The sum of fractions 5, 6 and 7 (equivalent to beta-1, beta-2, and beta-3 fractions of the beta-globulin migrating fraction), was the largest electrophoretogram globulin fraction in nursehounds (median 55.3%, 13.16 g/l), consistent with previous studies in other shark species such as the sand tiger shark which reported median 61.40% and 35.0 g/l values for beta-globulins. Undulate skates showed lower values in the beta-globulin migrating fraction (the sum of fractions 4, 5 and 6; equivalent to beta-1, beta-2 and beta-3 fractions), with median 41.30% and 12.19 g/l; these values were also lower than those previously reported in other batoid species such as the cownose ray (median 68.4%, 38.3 g/l). In mammals, beta-globulin fraction is mainly composed of transferrin and beta-lipoproteins, though can also include immunoglobulins, complement proteins, and acute phase proteins. In previous studies with elasmobranchs, proteins migrating in the fraction equivalent to beta-globulins have been hypothesized to be very-low-density lipoproteins and low-density lipoproteins [11, 22, 32].

The last fraction (fraction 7 in skates and fraction 8 in sharks) was well-defined in both species, migrated in the mammalian gamma-globulin fraction range, and consisted of median 1.90% and 0.54 g/l in undulate skates and 5.40% and 1.29 g/l in nursehounds. This fraction seems to be more preserved and showed similar levels across the different elasmobranch species, with 6.67% and 0.38 g/l in the sand tiger shark, 4.35% and 1.00 g/l in the Atlantic sharpnose shark, 3.60% and 1.00 g/l in the bonnethead shark and 4.76% and 1 g/l in the spiny dogfish [5, 31]. As demonstrated in previous studies with fish (including elasmobranch), this band may be useful to identify the immunoglobulin M response in this group of animals [3, 33].

Our results also showed significant interspecific differences between the two studied elasmobranch species when electrophoretograms were classified as four fractions (migrating in the regions of prealbumin, alpha-, beta-, and gamma-globulins) (P < 0.01; Mann-Whitney U test). This finding agrees with previous articles, which also report statistically significant interspecific differences for fractions 1, 2, 3, 4 and 5 between the Atlantic sharpnose shark, the bonnethead shark, and the spiny dogfish [5]. The marked interspecific differences for plasma protein and PPE fractions baseline values showed in this study possibly reflect species-specific differences in lipoprotein composition in elasmobranchs, revealing the need for further PPE studies involving different orders and families of elasmobranchs using the same methodology (capillary zone electrophoresis) to allow for a more accurate interspecific comparison and a reliable electrophoretogram interpretation in this group of animals.

Our study shows statistically significant differences (*p* < 0.01; Mann-Whitney U test) for alpha-2-/beta-globulin ratio between the undulate skate (median 1.01) and the nursehound (median 0.67). These interspecific differences coincide with previous studies performed with elasmobranchs, which also showed important variations for bonnethead and nurse shark fraction 3:4 ratio (equivalent to alpha-2-/beta-globulin ratio) in samples obtained from clinically healthy bonnethead sharks (mean 0.78) and clinically healthy nurse sharks (mean 0.23) [4, 22]. Previous studies performed with elasmobranchs revealed variations in fraction 3 (equivalent to alpha-2-globulins) linked to different clinical conditions, such as a decrease observed in bonnethead sharks with inflammatory pathologies. The ratio of fraction 3 to fraction 4 also decreased significantly in clinically unhealthy bonnetheads in comparison to clinically healthy individuals [4]. Because of this, further PPE studies with elasmobranchs should focus in providing species-specific values for this ratio, as well as analyzing its clinical use for the health evaluation and the disease diagnosis in this group of animals.

It must be taken into account that both species evaluated in this study are benthic, have a similar metabolism, behavior, reproduction (oviparous), and geographical distribution [13, 14, 16, 17]. In addition, all animals included in this study had been maintained under human care in very similar environmental conditions, all samples have been collected by the same team of professionals and have been processed in the same laboratory following the same methodology. Because of this, the significant differences observed between the undulate skate and the nursehound in this study suggest that greater differences can be expected in the PPE of pelagic elasmobranch species, animals from tropical regions at different water temperatures and metabolic rates, free-living populations with highly variable environmental and physiological conditions (including reproductive status), animals suffering from different pathologic conditions, as well as samples collected using different sampling techniques and sample processing protocols. Despite previous studies with avian plasma have demonstrated that a single freeze-thaw cycle does not produce statistically significant changes in PPE fractions, there are currently no studies evaluating the effect of freezing on elasmobranch PPE analysis [21]. A limitation to this study is that the impact of freezing could not be determined, and it is possible that the freeze-thaw cycle to which plasma samples were exposed affected the electrophoretograms. Further studies evaluating the impact of different freezing temperatures, periods, and freeze-thaw samples in aquatic animal species are needed to precisely interpret plasma protein electrophoretograms.

Another limitation to our study was the lack of a large number of diseased undulate skates and nursehounds for statistical comparison with the healthy sample population used for these species. However, the objective of this study was not to evaluate variations in the electrophoretogram associated to illness, but to determine baseline values in clinically healthy individuals. Further studies including much larger sample sizes of undulate skate and nursehounds under various pathological processes should study the causes of variation in the alpha-2 globulin region (fraction 3 in undulate skates and fraction 3 and 4 in nursehounds), as well as Alpha-2-/Beta-globulin ratio, which will allow for a more accurate interpretation of electropherogram variations associated with different diseases in these species.

In our study, no statistically significant differences were detected between sexes and developmental stages in the undulate skate, although statistically significant differences were detected in nursehounds: fraction 4% was significantly higher in males (median 27.58%) than in females (median 26.85%) (*p* = 0.03; Mann-Whitney U test). However, this difference, even if statistically relevant, remains very tiny and even not relevant on concentration values. Despite variations in some electrophoretogram fractions have been related to sex and season in humans and domestic animal species, previous PPE studies with elasmobranchs did not detect statistically significant variations related to sex in different shark species including the blacknose, the blacktip, the nurse, bull, lemon, sandbar, and the tiger shark [11, 22, 23]. Fraction 4 (equivalent to beta-globulins) resulted significantly higher in female bamboo shark compared to male individuals, though the study did not determine shark reproductive status and the protein composition of this fraction [9]. It should be also considered that, in our study, all undulate skates were sampled during the same season (February 2021 – winter), while all sharks were sampled during August 2021 – summer, and therefore the effect of season could not be evaluated for each species. Another point to consider is that most of the skates sampled in this study were juveniles. As such, it was not possible to compare variations between sexes in the adult skate population due to the low number of clinically healthy adult skates available for sampling. In addition, statistically significant variations were also detected in nursehounds between developmental stages in fractions 5 (*p* = 0.033; Mann-Whitney U test), 6 (*p* < 0.01; Mann-Whitney U test) and 8 (*p* < 0.01; Student’s t-test) percentages. Three out of the four fractions showing statistically significant differences in nursehounds (fractions 4, 5 and 6) belonged to the proteins migrating in the beta-globulin region (equivalent to beta-1, beta-2, and beta-3 globulin fractions). Beta-globulins consist of multiple fractions, which include sex-hormone-binding globulins, and which have been described to vary between the different developmental and reproductive stages with reported statistically significant differences between male and female bamboo sharks [9]. Furthermore, the remaining fraction showing statistically significant differences in nursehound (fraction 8), corresponded to proteins migrating in the gamma-globulin region, which has been considered to represent IgM in teleost and has also been described to vary significantly not only during infection, but also with age and season in different animal species [11]. While our study followed ASVCP guidelines for the determination of reference values in clinically healthy populations, it is important to consider that within the few PPE studies performed with elasmobranchs, there is sometimes a mix of healthy and diseased individuals, which should be reflected and considered as this can significantly influence the reliability of the values provided [3–5, 9, 22, 23]. Understanding which electrophoretic changes are related with sex or developmental stage in elasmobranchs is key for the correct interpretation of plasma protein fraction values and to differentiate which abnormalities are due to ongoing pathologic processes or to physiological variations. Despite the potential of PPE as an analytical tool for health evaluation in elasmobranchs, further detailed studies are needed to better understand the electrophoretic patterns during elasmobranch development, physiological states, between sexes, and most importantly, during illness.

## Conclusion

The baseline values provided in this study for clinically healthy undulate skates and nursehound sharks will contribute to the limited data available on elasmobranch plasma protein electrophoresis analysis, and thus will aid in the clinical evaluation and health management of elasmobranchs. Further studies including a larger number of undulate skates and nursehounds suffering from different pathologies will help further our understanding and more accurate interpretation of plasma protein electrophoretograms for these species. In addition, due to the significant variations observed in PPE across elasmobranch species, further studies involving different elasmobranch species are necessary for a reliable interspecific electrophoretogram comparison and interpretation.

## Data Availability

Data used to develop this study is available upon request to the authors.
